# Free-Ranging Farm Cats: Home Range Size and Predation on a Livestock Unit In Northwest Georgia

**DOI:** 10.1371/journal.pone.0120513

**Published:** 2015-04-20

**Authors:** Susanna E. Kitts-Morgan, Kyle C. Caires, Lisa A. Bohannon, Elizabeth I. Parsons, Katharine A. Hilburn

**Affiliations:** 1 Department of Animal Science, Berry College, Mount Berry, Georgia, United States of America; 2 Department of Biology, Berry College, Mount Berry, Georgia, United States of America; University of Regina, CANADA

## Abstract

This study’s objective was to determine seasonal and diurnal vs. nocturnal home range size, as well as predation for free-ranging farm cats at a livestock unit in Northwest Georgia. Seven adult cats were tracked with attached GPS units for up to two weeks for one spring and two summer seasons from May 2010 through August 2011. Three and five cats were tracked for up to two weeks during the fall and winter seasons, respectively. Feline scat was collected during this entire period. Cats were fed a commercial cat food daily. There was no seasonal effect (P > 0.05) on overall (95% KDE and 90% KDE) or core home range size (50% KDE). Male cats tended (P = 0.08) to have larger diurnal and nocturnal core home ranges (1.09 ha) compared to female cats (0.64 ha). Reproductively intact cats (n = 2) had larger (P < 0.0001) diurnal and nocturnal home ranges as compared to altered cats. Feline scat processing separated scat into prey parts, and of the 210 feline scats collected during the study, 75.24% contained hair. Of these 158 scat samples, 86 contained non-cat hair and 72 contained only cat hair. Other prey components included fragments of bone in 21.43% of scat and teeth in 12.86% of scat. Teeth were used to identify mammalian prey hunted by these cats, of which the Hispid cotton rat (*Sigmodon hispidus*) was the primary rodent. Other targeted mammals were *Peromyscus sp*., *Sylvilagus sp*. and *Microtus sp*. Invertebrates and birds were less important as prey, but all mammalian prey identified in this study consisted of native animals. While the free-ranging farm cats in this study did not adjust their home range seasonally, sex and reproductive status did increase diurnal and nocturnal home range size. Ultimately, larger home ranges of free-ranging cats could negatively impact native wildlife.

## Introduction

*Felis silvestris catus*, the modern day domestic cat, is the most widespread terrestrial carnivorous species on earth and as such, can have a major impact on native prey populations, including small mammals, avian species, and insects [1]. In the past 40 years, the number of domestic cats in the United States has tripled [2]. Regardless of how cats are classified (inside/outside house cats, free-ranging, feral or semi-feral), it is possible for them to have negative effects on native wildlife populations due to their predatory nature. Furthermore, feral and free-ranging cats can be present in very high densities, which can potentially lead to devastating effects on native animals. Research in Hawaii has shown feral cats to be frequent predators of endangered Hawaiian birds [3, 4, 5]. Annual mammal mortality due to cats in the United States has been estimated to be between 6.9 and 20.7 billion, with 89% of the mortality caused by un-owned cats, including farm/barn cats [6]. Research has also suggested that farm/barn cats living at higher densities and receiving some food from humans and can have substantial effects on local native animal populations [7]. Cats in these situations can be described as subsidized predators, affecting not only native prey, but possibly out-competing native predators. Research also supports that regular access to food provided by humans does not suppress hunting behavior in cats, and they may continue to kill even when prey populations are low [8, 9].

Some of the native mammals available as prey to cats in Northwest Georgia include animals from the Orders Insectivora, Lagomorpha, and Rodentia [10]. Additionally, non-native rodents such as the black rat (*Rattus rattus*), Norway rat (*Rattus norvegicus*), and house mouse (*Mus musculus*) are commonly associated with human activity and buildings [11]. The habitat for these animals, along with birds, invertebrates and reptiles, is extensive in agricultural settings, as there are usually barns, fields and possibly streams. While free-ranging cats may have a beneficial role in controlling the non-native rodents on a farm, they can exert extreme pressure on native wildlife as well [12]. Cats are also known to prey on other animals such as invertebrates, reptiles, amphibians and birds [13, 14]. However, prey opportunities differ with geographical and ecological regions, i.e., cats on islands have been implicated in local extinction of birds and reptiles [15, 16].

It is important not only to determine the prey targeted by cats, but also to understand their home range and factors that affect their tenancy. Mapping the home range of cats can determine how far they roam and the areas in which they roam, hunt, etc. In free-ranging cats, whether the animal is sterilized or intact could possibly affect the size of its home range, and subsequently, the extent of its impact on native wildlife. Some research has shown that sterilized male and female cats have similar-sized home ranges, and cats living within the same territory overlap in core area but differ in the size of outer home ranges [17]. Sex of the cat may also affect size of the home range. Feral male cats have been shown to have consistently larger home ranges as compared to females [18], but it is possible for both males and females to have large home ranges based on their reproductive and nutritional status, and season of the year, so these relationships remain poorly understood.

A great deal of research has studied home range size and diet of free-ranging cats in various areas of the world; however, less has been done to study predation and home range size in free-ranging farm cats within the United States, particularly the Southeast. The overall purpose of the current research was to assess hunting of wildlife by seven, free-ranging farm cats located at the Gunby equine center on the Berry College campus. To accomplish this goal, we established the seasonal, diurnal and nocturnal home ranges of these cats, and assessed the prey hunted within those home ranges using feline scat analysis. We hypothesized that cats residing in a fixed area would occupy different-sized home range territories according to season, day and night, and that sex and reproductive status of these cats would affect home range size. We also hypothesized that the primary prey of these cats would consist of rodents.

## Materials and Methods

### Ethics Statement

The protocol for this study and all animal handling techniques were approved by the Berry College Institutional Animal Care and Use Committee (Permit Number: 2009/10-004). The study was conducted on the Berry College campus in and around the Gunby equine center (34°3’ N, 85°2’ W). This land is privately owned by Berry College and no specific permission was required for this study. No endangered or protected species were involved in this study. Small mammals live-trapped in this study were collected under Scientific Permit Number: 12532 issued by the Georgia Department of Natural Resources, Special Permit Unit.

### Study Area

The study area evaluated for cat home ranges and used for feline scat collections was located in Northwest Georgia, and consisted of approximately 44 hectares located within 8 km of Rome, Georgia. The study was conducted on the Berry College campus in and around the Gunby equine center (34°3’ N, 85°2’ W). Topography consists of low rolling hills within open pastures, as well as woods and a natural riparian zone fenced to exclude horses. Within the study site, the average width of the riparian zone is 15 meters and the length is approximately 1.2 km. Also located within part of the study site area is a covered arena, student housing, barns, horse lots, outdoor riding areas, as well as paved and gravel roads leading into and through the Equine Center. The climate in this region is characterized by hot, humid summers and mild winters. During the period of the current study (May, 2010 – August, 2011), the average annual precipitation was 124.91 cm and average annual temperature was 19.8° C (Georgia Automated Environmental Monitoring Network).

### Cats and GPS Tracking

All cats used in the study were known to primarily reside at the Gunby equine center and surrounding areas. These cats were considered to be free-ranging, as they did not enter human dwellings, but were fed and cared for by humans. Seven physiologically mature cats (5 female, 2 male; Table 1) were fitted with a typical cat harness acquired from a local pet store. To ensure the safety of the animals, cat harnesses were modified to break if cats became caught and immobilized. Cats were acclimated to the harnesses for up to one month prior to the beginning of the study. For tracking movement of the cats, CatTraQ GPS units (44 x 27 x 18 mm; 22g; Perthold Engineering LLC, Anderson, SC) were attached to the harness. These GPS units contain a SkyTraq Venus 5 chip, which has an accuracy of 5m CEP. To gain an adequate number of GPS coordinates, each cat remained harnessed for up to two weeks during the spring, summer, fall and winter of 2010–2011. While a cat was harnessed, the units were programmed to obtain GPS coordinates every 30 minutes. Once harnessed, cats were checked daily to ensure that the harness and GPS unit were secure and functioning properly. Cats were also fed a commercial cat food diet daily. Cats were generally offered food in the mornings at approximately 0700 and it is estimated that approximately a total of 200 g of cat food was offered (~30 g/cat). The amount of food offered during the study was not manipulated or altered from the established feeding schedule. It was conveyed to us by the unit supervisor that the cats were fed only small amounts of cat food daily to encourage them to hunt and keep the buildings free of rodents. Between May 2010, and August 2011, a total of 5,904 GPS fixes were obtained on the free-ranging cats residing at the Gunby Equine Center.

**Table 1:** Sex, reproductive status and approximate ages of cats used to determine home range on the Berry College campus (2010–2011).

Cat name	Sex	Reproductive status	Approximate age (yrs)
Cat 1	Female	Intact	6
Cat 2	Female	Spayed	10
Cat 3	Female	Spayed	6
Cat 4	Female	Spayed	4
Cat 5	Female	Spayed	7
Cat 6	Male	Intact	3
Cat 7	Male	Neutered	13

### Home Range Analyses

The Geospatial Modelling Environment [19], the statistical software R [20], and ESRI ArcMap 10.0 [21] were used to calculate home range areas (95%, 90% and 50% probability areas) using a fixed kernel estimator (KDE). Home-range area was calculated from 95% and 90% of the GPS coordinates for each animal (95% and 90% KDE), assuming that the remaining 5% and 10% of coordinates, as determined by the software, were outliers that represented excursions. Core home range, defined as the area used disproportionately more than other areas [22], was estimated using 50% KDE. The plug-in estimator was used to estimate a kernel smoothing parameter for each cat and a cell size of 10. Home ranges were calculated for 5 seasons: summer 2010 (May 17-Sep 30, 2010), fall (Oct 1-Dec 22, 2010), winter (Dec 23, 2010-Feb 27, 2011), spring (Mar 1-Apr 30, 2011), summer 2011 (May 1-Aug 13, 2011). The entire month of March was included in the spring home-range calculation, as the weather was more spring-like than winter-like (avg. temp = 12.12° C). Likewise, the entire months of May and June are included in the summer home-range calculations, as the weather was summer-like during these months (avg. temp = 23.21° C). Home ranges for each cat based on diurnal/nocturnal movement were also calculated.

### Feline scat and hair analyses

Throughout the study, feline scat samples were collected daily from known latrine areas within the identified home range area of the 7 free-ranging cats. Five primary latrine areas were identified, as well as 3 other areas where feline scat was occasionally found. All of these areas were within 150 m of the feeding area for the cats. Prior to commencement of this study, all participating cats were observed and confirmed to use one or more of these latrine areas. All cats were also confirmed to hunt, as they were observed with prey items at various times by barn and research workers. Between May 2010 and August 2011, 210 scat samples were collected and stored at -20°C until time of processing. At time of processing, samples were dried for 3 d at 60° C in a drying oven and then carefully separated into different components, including fecal material, hair, invertebrates, plant material, bones/teeth, rock, sand, feathers and other material. Percentage occurrence of an item was defined as the proportion of scats that contained that particular item. As many scats contained more than one type of prey item, the sum of percentage occurrences could exceed 100%.

If possible, feline scat samples containing teeth were analyzed to identify mammalian prey species. For teeth identification, we consulted experts at the Jones Ecological Research Center (Newton, GA) and study skins of small mammals at the Georgia Museum of Natural History (Athens, GA). In samples containing no teeth, hair was analyzed to classify as either “cat” or “non-cat.” To assist in identification of hair as “cat” or “non-cat,” hair samples of all cats included in the study were collected, as well as hair from small mammals live-trapped within the home range area. Hair samples were obtained from *P*. *maniculatus*, *R*. *humilis*, *N*. *floridana*, *M*. *musculus*, *S*. *hispidus*, *B*. *carolinensis*, *M*. *pennsylvanicus and T*. *striatus*. Because the hair of most mammals generally contains two types of hair, guard hairs in the overcoat and fine hairs in the undercoat, both types of hair were collected from the known cats and small mammals [23]. Samples of all known hairs were permanently mounted on microscope slides using VectaMount AQ (Vector Labs, Burlingame, CA, USA) to achieve an optimal refractive index for analyses. At least 7–10 individual hairs, including guard and fine hairs, were randomly selected from each scat sample to examine. Hairs were gently washed with water, temporarily mounted on a microscope slide and examined using a compound light microscope under 100x and 400x magnification. Hairs were then determined to be either “cat” or “non-cat.”

### Statistical Analyses

All datasets are presented as the mean ± SEM and differences between means were considered significant at *P < 0*.*05*. As previously mentioned, home range estimates were calculated using kernel density estimation (KDE) at 95%, 90% and 50% isopleths for all individuals (n > 6). The main effects and interactions between sex, endocrine status (intact vs. sterilized), time of day (diurnal vs. nocturnal), and season on home range size at multiple isopleths was evaluated using two-way ANOVA with post-hoc comparisons using Bonferroni-Holm method. Seasonal effects on home range size were calculated using one-way ANOVA and pairwise comparisons were evaluated with a Newman-Keuls multiple-range test.

## Results

The home range estimates for 95, 90 and 50% kernel density estimations (KDE) for the seven free-ranging cats in the study are presented and unfortunately, due either to temporary loss of harnesses with attached GPS units or injuries, we were unable to collect data on all 7 cats during the fall and winter seasons (Table 2). Over the course of the study, home range sizes ranged from 4.26 ha to 10.23 ha at 95% KDE for all cats. While there were no differences in the 95, 90 or 50% KDE home ranges for all cats (*P* > 0.05), numerically, the winter home range was the largest at 95, 90 and 50% KDE (Table 2). The spring and summer 2011 home ranges were numerically smaller at 95 and 90% KDE, while fall was smallest at 50% KDE (Table 2).

**Table 2:** Home range estimates (ha; 95% kernel density estimation [KDE], 90% KDE, 50% KDE) for free-ranging domestic cats in Mount Berry, Georgia, USA across seasons.

		95% KDE		90% KDE		50% KDE	
Season	*n*	Mean	SEM	Mean	SEM	Mean	SEM
***All cats***							
Summer 2010	7	5.27	1.42	3.70	0.99	0.79	0.23
Fall 2010	3	5.52	1.54	3.97	1.13	0.67	0.16
Winter 2011	5	10.23	5.96	7.31	4.20	1.36	0.70
Spring 2011	7	4.26	1.13	3.22	0.87	0.81	0.22
Summer 2011	7	4.85	1.10	3.21	0.70	0.53	0.10
***Altered cats***							
Summer 2010	5	3.20	0.33	2.24	0.23	0.49	0.06
Fall 2010	3	5.52	1.54	3.97	1.13	0.67	0.16
Winter 2011	3	3.70	0.39	2.63	0.25	0.56	0.02
Spring 2011	5	2.66	0.67	1.99	0.51	0.48	0.12
Summer 2011	5	3.45	0.48	2.32	0.28	0.46	0.05

The reproductively intact cats (Cat 1 and Cat 6) were also removed from the data to show seasonal home range sizes for only sterilized cats (Table 2). Once these cats were removed from the data set, not only did the home range sizes numerically decrease at all isopleths, but the range in size among the seasonal home ranges decreased as well. When the reproductively intact cats were included in the data set (Table 2), the home ranges at 95% KDE spanned from 4.26 to 10.23 ha. When these cats were removed from the data set (Table 2), the range was only 2.66 to 5.52 ha at 95% KDE. Among the sterilized cats at 95 and 90% KDE, fall home range was the largest, while spring home range was the smallest (Table 2). At 50% KDE, fall home range is also the largest, but summer 2011 home range is the smallest (Table 2).

**Fig 1:**
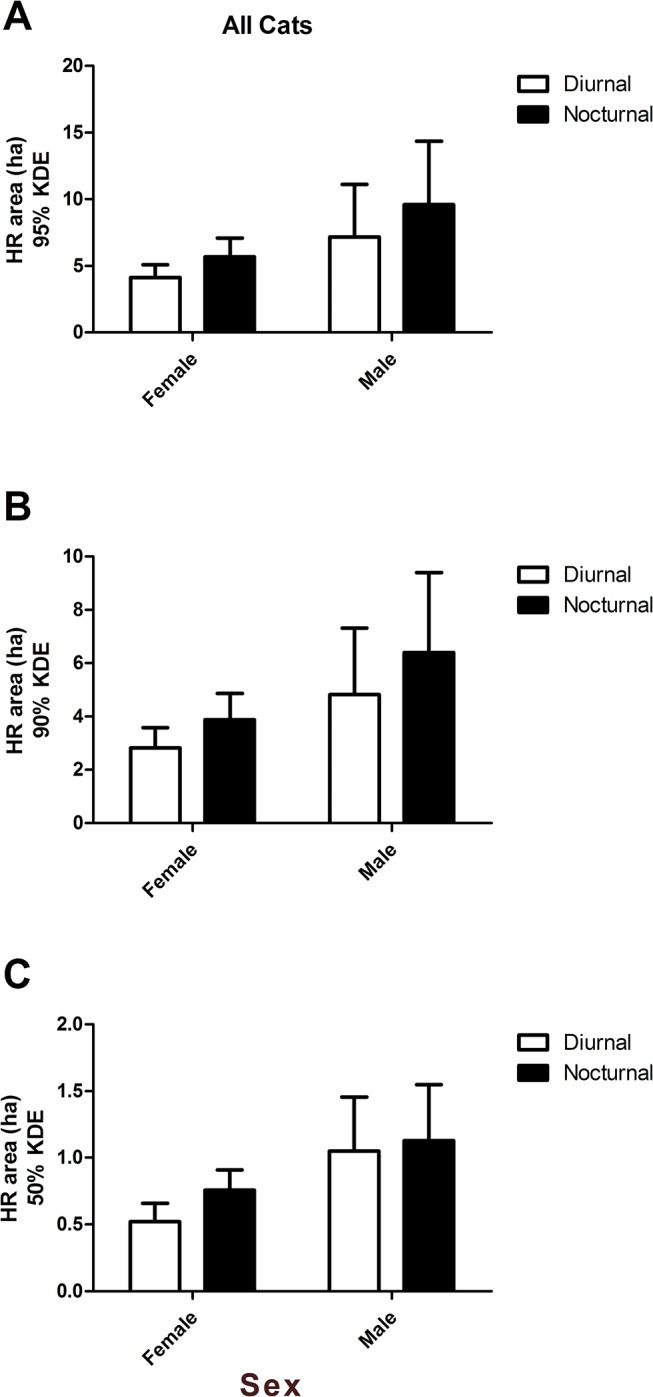
Diurnal and nocturnal home ranges for male and female free-ranging cats. Panel A includes diurnal and nocturnal home range (HR) area in hectares (ha) at 95% kernel density estimation (KDE) for male and female cats, Panel B includes diurnal and nocturnal HR area (ha) at 90% KDE for male and female cats, and Panel C includes diurnal and nocturnal HR area (ha) at 50% KDE for male and female cats.

**Fig 2:**
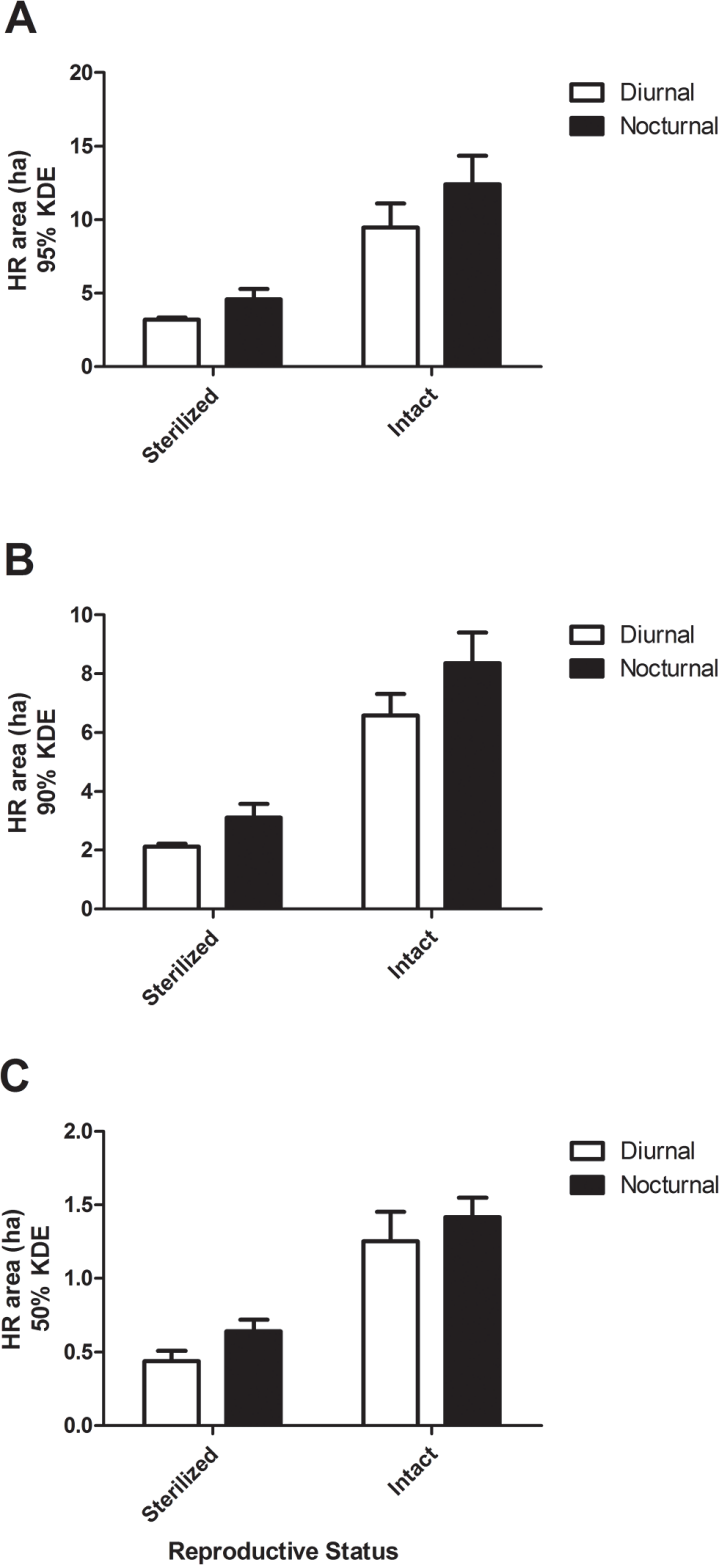
Diurnal and nocturnal home ranges for reproductively intact and sterilized free-ranging cats. Panel A includes diurnal and nocturnal home range (HR) area in hectares (ha) at 95% kernel density estimation (KDE) for intact and sterilized cats, Panel B includes diurnal and nocturnal HR area (ha) at 90% KDE for intact and sterilized cats, and Panel C includes diurnal and nocturnal HR area (ha) at 50% KDE for intact and sterilized cats.

While there were no differences in diurnal vs. nocturnal home range sizes at 95% KDE (*P* > 0.05), nocturnal home range was larger for all cats at 90% KDE (*P* = 0.02) and at 50% KDE (*P* = 0.03; Table 3). When we analyzed the effects of sex on diurnal and nocturnal home range size, there was no effect of sex at 95 and 90% KDE (*P* > 0.05; Fig 1). At 50% KDE, male cats had numerically larger (*P* = 0.08) diurnal and nocturnal (1.05 and 1.13 ha, respectively) core home ranges as compared to female cats (0.52 and 0.76 ha, respectively). The large amount of variation associated with the diurnal and nocturnal home range estimates of the male cats was largely due to the intact male (Cat 6), who had home ranges 3–3.5 times the size of the sterilized male cat (Cat 7). Within male or female cats, there were no differences in the size of diurnal vs. nocturnal home range (*P* > 0.05). The effect of reproductive status on diurnal and nocturnal home range size was the most striking result of this study. At 95, 90 and 50% KDE, reproductively intact cats had larger (*P* < 0.0001) diurnal and nocturnal home ranges as compared to sterilized cats (Fig 2). Also, within both intact and sterilized cats, nocturnal home range was larger than diurnal home range for the overall home range at 95 (*P* = 0.04) and 90% KDE (*P* = 0.03).

**Table 3:** Home range estimates (ha; 95% kernel density estimation [KDE], 90% KDE, 50% KDE) for free-ranging domestic cats in Mount Berry, Georgia, USA for diurnal vs. nocturnal movement.

		95% KDE		90% KDE		50% KDE	
Period	*n*	Mean	SEM	Mean	SEM	Mean	SEM
***All cats***							
Diurnal	7	5.00	1.21	3.40^a^	0.84	0.67^a^	0.16
Nocturnal	7	6.81	1.59	4.61^b^	1.05	0.86^b^	0.16

^a,b^Means with different superscripts differ, *P ≤ 0*.*05*.

Over the course of the study, we collected 210 feline scats from within the home range of the free-ranging cats. Non-prey material (undigested cat food and endogenous secretions) was found in 91.43% of the scat samples collected, and hair was the next most dominant component identified in 75.24% of samples (Table 4). Of the samples containing hair, 54.43% were identified as non-cat hair, while 45.57% were identified as predominantly cat hair (Table 4). Invertebrates were also found in 41.9% of the scat samples, while feathers were only found in 2.38% (Table 4). Lastly, bones and teeth were only found in 21.43% and 12.86% of the scat samples, respectively (Table 4). The samples containing teeth allowed us to identify at least the genus or genus + species of the prey killed by the cats (Table 5). The dominant prey identified were Hispid cotton rat (*Sigmodon hispidus*), followed by *Peromyscus sp*. (Table 5). We also identified two or fewer each of rabbit (*Sylvilagus sp*.), vole (*Microtus sp*.), and Eastern woodrat (*N*. *floridana*; Table 5). In five of the samples, we found hollow bones and feathers, which indicated avian species (Table 5).

**Table 4:** Percentages of 210 feline scats containing prey components.

Scat component	% of scats containing item
Non-prey material	91.43
Hair	75.24
Non-cat hair	54.43
Cat hair	45.57
Invertebrates	41.90
Bones	21.43
Teeth	12.86
Feathers	2.38

Samples were collected May, 2010, through August, 2011, for free-ranging domestic cats in Mount Berry, Georgia, USA.

**Table 5:** Number of prey identified in 27 feline scats containing prey teeth or feathers and collected May, 2010, through August, 2011, from free-ranging domestic cats in Mount Berry, Georgia, USA.

Species	Number identified
*Peromyscus sp*.	6
*Sigmodon hispidus*	11
*Sylvilagus sp*.	2
*Microtus sp*.	2
*N*. *floridana*	1
Avian sp.	5

## Discussion

The objective of this study was to assess the hunting of wildlife by a population of free-ranging cats both by establishing their home-range area and identifying prey in feline scat samples. We wanted to gain a better understanding of hunting by these cats, so measuring home ranges allowed us to determine the size of the area in which they hunted. Furthermore, by evaluating seasonal and diurnal/nocturnal home ranges, we were able to discover if the cats expanded their home range during certain times of the year or if they preferred to roam/hunt during the day or night. We also wanted to know if the cats were targeting particular wildlife such as invertebrates, birds, or small mammals; thus, scat analysis gave us some insight into their prey preferences. The equine center on the Berry College campus provided an excellent area in which to study these cats, as both natural and man-made habitats exist, thus providing plenty of areas for both native and non-native wildlife (prey) to reside. While our population of cats provided a small sample size of only 7 cats, other studies have used similar numbers of cats (usually less than 10 cats) to study home range and diet [1, 17, 24, 25, 26].

We chose to evaluate home ranges at all three isopleths to observe the gradient of decrease over a season, the size of the overall home range (95% KDE), and the core home range (50% KDE). When we evaluated the home ranges of all cats or only sterilized cats in this study (Table 2), we saw no differences between seasons at 95, 90 or 50% KDE. Seasonal home range size at 95% KDE for all cats was 4.26–10.23 ha, and 2.66–5.52 ha for sterilized cats. Other research has found home ranges of free-ranging cats to be highly variable, extending from 0.04–228 ha [17, 25, 27, 28]. It is not surprising that the home ranges (95% KDE) of the cats in our study were smaller as they were habituated to humans and received small amounts of cat food daily (approximately 30 g/cat), which may have influenced the distance they roamed away from the barns and other buildings. Indeed, the core home range of these cats (50% KDE) was very small (0.53–1.36 ha) and generally centered over the common feeding area in one of the barns. We noted that for all cats across seasons, core home ranges and overall home ranges (95% KDE) overlapped, regardless of sex or reproductive status. This overlapping is most likely due to the common feeding area and shelter available in the barns, as other research suggests that free-ranging cats spend most of their time where food and shelter are available [29].

When all cats are considered, the numerically larger home range size during the winter season (10.23 ha, 95% KDE) is likely due to the inclusion of the two reproductively intact cats, as this home range falls into a similar range as the other seasons when those cats are removed (3.7 ha, 95% KDE; Table 2). It is unclear why these cats roamed farther during the winter months. It is possible they were searching for mates, as domestic cats are certainly capable of reproducing during any month of the year [30, 31]. However, other research suggests that ovarian activity ceases in the female domestic cat with decreasing photoperiod, and male cats show somewhat decreased spermatogenic activity during winter months as well [32, 33].

We observed both the diurnal and nocturnal home ranges of reproductively intact cats to be over twice as large as those of sterilized cats at 95, 90 and 50% KDE. While we must caution that our sample size of intact cats was only two, it is still remarkable that a difference in home range size between five sterilized and two intact cats was so obvious. Other research has mainly studied the effects of reproductive status on annual or seasonal home range size using feral cats, not free-ranging cats [18]. In the study by Guttilla and Stapp [18], there were no differences in home range size between sterilized and intact feral cats. In another study investigating home ranges in free-roaming cats in the Chicago metropolitan area, reproductive status had no effect on home range size [34]. We also found that within both sterilized and intact cats at 95 and 90% KDE, nocturnal home range was larger than diurnal home range (Fig 2). Other research has found that among de-sexed suburban cats, no differences occurred in the size of diurnal vs. nocturnal home ranges, but nocturnal was larger than diurnal home range for intact farm cats [17]. The cats in our study may have simply preferred to hunt at night instead of during the day, when they may have remained closer to or inside the barns. We also assessed the effect of sex on day and night home range size and found that males tended to have larger core (50% KDE) day and night home ranges. Other research also found feral male home ranges (50% KDE) to be larger than females [18, 34].

While studying and mapping the home ranges of our cats, we also identified feline latrine areas, mostly located in and around the barns and buildings. Prior to and during the study, we regularly observed all of the cats using these latrine areas. While we recognize that the cats may have occasionally used areas outside of the known latrine areas and we did not find those scats, we do believe that we recovered a representative sample from all cats within the known latrine areas. Over the course of the study, we collected feline scat in an effort to determine the wildlife targeted by these cats. Not surprisingly, the majority of the scat collected contained non-prey material (undigested food and endogenous secretions), which is a result of the dry cat food these cats received daily and illustrates their dependence on humans for food. The next most abundant item in the feline scat was hair, which consisted of both cat hair ingested from grooming and hair from prey remains. Hair examination revealed more about the cats’ hunting behavior with regard to small mammals, as less than 25% of the scat collected contained bones or teeth. Indeed, of 158 scat samples containing hair, 86 of those contained non-cat hair. While we did not attempt to identify species using the hair, we noted that the majority resembled our known rodent hairs. It is important to note that only 45 scat samples contained small amounts of fragmented small mammal or bird bones and only 28 of those samples contained teeth. Therefore, an additional 41 scat samples contained non-cat hair but no other prey parts, which suggests catching/killing of prey by the cats, but very little or no consumption of the kill. Indeed, in another study investigating predation of free-roaming cats, only 28% of prey items were consumed [35]. Our findings may also support the notion that while these cats received some daily dry cat food, their hunting instinct was not suppressed and while they killed prey, they were not hungry enough to completely consume it. This idea is supported by other research which indicates that regular access to cat food does not subdue hunting behavior in farm cats [9].

In the scat samples containing teeth, we were able to identify either genus or genus and species of the prey consumed. Because the teeth within some genera such as *Peromyscus*, *Sylvilagus*, and *Microtus* are so similar, it is not always possible to distinguish between species. Hispid cotton rat (*Sigmodon hispidus*) was the dominant small mammal identified in the scat, which is not surprising considering it is likely the most abundant native mammal in Georgia and is found state-wide [10]. *Peromyscus sp*. were also identified in the scat, which could include the American deer mouse (*Peromyscus maniculatus*), white-footed mouse (*Peromyscus leucopus*), or cotton mouse (*Peromyscus gossypinus*), all of which are species found in Northwest Georgia [10]. Other mammals identified were rabbit (*Sylvilagus sp*.), vole (*Microtus sp*.*)* and Eastern woodrat (*N*. *floridana*). Possible rabbit species found in Northwest Georgia are the swamp rabbit (*Sylvilagus aquaticus*) and Eastern cottontail (*Sylvilagus floridanus*; [10]). It is likely that the cats killed juvenile or small adult rabbits, as any of the possible species killed could reach 0.9–2.3 kg as adults. Other research has shown that cats tend not to kill prey larger than about half the size of the cat itself [36]. Two species of voles are found in Northwest Georgia – meadow vole (*Microtus pennsylvanicus*) and woodland vole (*Microtus pinetorum*) – and could be preyed upon by cats [10]. While all of these mammals probably represent most of the species hunted by our free-ranging cats, the total number of prey killed or killed and consumed during this study would be difficult to determine, as we likely did not find and collect all of the feline scat. However, based on teeth and hair analyses, it is clear that rodent species were the primary prey taxon taken by these free-ranging cats. Research by Mitchell and Beck [36] also showed that rodent species were primary prey of free-ranging cats. Furthermore, the presence of a riparian area within the home range of our cats provided an excellent habitat for small mammals, as it is fenced to exclude horses while grass, trees and shrubs grow uninhibited. We believe the majority of hunting by the cats occurred in this area, as GPS data showed them in this area a great deal and we have live-trapped small mammals in this area (data not shown).

While we were also able to use fragmented bones, some of which contained attached feathers, to identify avian species in the feline scat, it is difficult to determine the extent to which our cats targeted birds. In other studies, birds generally rank behind rodents and/or lagomorphs in prey taxons hunted by cats (Mitchell and Beck, [36]). This is likely because birds tend to become primary prey for cats only when mammalian prey becomes scarce, such as on islands [37]. However, it is also quite difficult to accurately determine predation of birds by domestic cats using scat, as research has shown that bird remains experience more fragmentation during digestion than do mammalian remains, which may underestimate consumption of birds [38]. While it is certainly possible that the cats in our study killed and consumed more birds than we were able to detect in their scat due to fragmentation, it is also likely that they didn’t hunt many birds because so many small mammals were available to them.

Invertebrates were present in 41.9% of the feline scats we collected, but we noted there were usually only 1–2 individuals per scat. Most of the invertebrates were grasshoppers or beetles, which is similar to other research [39]. Overall, invertebrates were likely less important in the diet of our free-ranging cats due to the higher availability of small mammals throughout the year. Also, while we did not evaluate feline scat by season, there was probably a seasonal decline in the availability of invertebrates throughout the winter months, as they would be less active during that time.

In this study, we were surprised not to find evidence of non-native rodents such as the house mouse (*Mus musculus*), Norway rat (*Rattus norvegicus*), or black rat (*Rattus rattus*) in any of the feline scat. These are commensal rodents, and therefore thrive in close proximity to humans [11, 40]. We would expect to find any of these species in the barns and buildings, and the house mouse can be found in any habitat of an agricultural setting [41]. However, it is likely that the cats eliminated any of these rodents from the buildings, as workers report that there are no problems with rats or mice in the barns. While house mice may not be residing in the barns or other buildings, we have live-trapped them in the riparian area, which indicates they have found a suitable alternative habitat to the barns. It is therefore probable that the cats would hunt them as well; we simply did not find any feline scat containing house mice remains.

This study assessed the hunting of free-ranging cats in and around a livestock unit in Northwest Georgia. We showed that while seasonal home range sizes for these cats remained relatively similar, diurnal and nocturnal home ranges were larger for reproductively intact cats. Cats with larger home ranges could potentially inflict more damage on wildlife. Feline scat analyses also showed that these cats hunted mainly native small mammals, most likely within a riparian area of their home range. If a large number of cats concentrate hunting within a small area, it is possible that the population of wildlife targeted within that area could become stressed. While rodents and lagomorphs are usually the preferred prey of cats, birds, invertebrates and reptiles are also hunted, depending on mammal availability and geographic location. As long as environmental conditions are suitable, these animals may be able to withstand intense hunting by cats and native predators. However, if their habitat is sufficiently disturbed and hunting pressure increases, it is possible that numbers of certain native species could decline.
